# Rescue of Vasopressin Synthesis in Magnocellular Neurons of the Supraoptic Nucleus Normalises Acute Stress-Induced Adrenocorticotropin Secretion and Unmasks an Effect on Social Behaviour in Male Vasopressin-Deficient Brattleboro Rats

**DOI:** 10.3390/ijms23031357

**Published:** 2022-01-25

**Authors:** Bibiána Török, Péter Csikota, Anna Fodor, Diána Balázsfi, Szilamér Ferenczi, Kornél Demeter, Zsuzsanna E. Tóth, Katalin Könczöl, Judith Camats Perna, Imre Farkas, Krisztina J. Kovács, József Haller, Mario Engelmann, Dóra Zelena

**Affiliations:** 1 Centre for Neuroscience, Szentágothai Research Centre, Institute of Physiology, Medical School, University of Pécs, 7624 Pécs, Hungary; torok.bibiana@gmail.com; 2 Laboratory of Behavioural and Stress Studies, Institute of Experimental Medicine, 1083 Budapest, Hungary; csikotapeter@gmail.com (P.C.); anna_@index.hu (A.F.); balazsfi.diana@gmail.com (D.B.); demeter@koki.hu (K.D.); Haller.Jozsef@uni-nke.hu (J.H.); 3 János Szentágothai School of Neurosciences, Semmelweis University, 1085 Budapest, Hungary; 4 Laboratory of Molecular Neuroendocrinology, Institute of Experimental Medicine, 1083 Budapest, Hungary; ferenczi.szilamer@koki.mta.hu (S.F.); kovacs.krisztina@koki.mta.hu (K.J.K.); 5 Department of Anatomy, Histology and Embryology, Semmelweis University, 1094 Budapest, Hungary; Tothzs@ana.sote.hu (Z.E.T.); konkat6@gmail.com (K.K.); 6 AG Neuroendokrinologie & Verhalten, Otto-von-Guericke-Universität Magdeburg, Institut für Biochemie und Zellbiologie, 39120 Magdeburg, Germany; j.camatsperna@uq.edu.au (J.C.P.); Mario.Engelmann@med.ovgu.de (M.E.); 7 Laboratory of Reproductive Neurobiology, Institute of Experimental Medicine, 1083 Budapest, Hungary; farkas.imre@koki.mta.hu; 8 Center for Behavioural Brain Sciences (CBBS), 39120 Magdeburg, Germany

**Keywords:** vasopressin, adrenocorticotropin, corticosterone, c-Fos, medial amygdala, social behaviour, supraoptic nucleus

## Abstract

The relevance of vasopressin (AVP) of magnocellular origin to the regulation of the endocrine stress axis and related behaviour is still under discussion. We aimed to obtain deeper insight into this process. To rescue magnocellular AVP synthesis, a vasopressin-containing adeno-associated virus vector (AVP-AAV) was injected into the supraoptic nucleus (SON) of AVP-deficient Brattleboro rats (di/di). We compared +/+, di/di, and AVP-AAV treated di/di male rats. The AVP-AAV treatment rescued the AVP synthesis in the SON both morphologically and functionally. It also rescued the peak of adrenocorticotropin release triggered by immune and metabolic challenges without affecting corticosterone levels. The elevated corticotropin-releasing hormone receptor 1 mRNA levels in the anterior pituitary of di/di-rats were diminished by the AVP-AAV-treatment. The altered c-Fos synthesis in di/di-rats in response to a metabolic stressor was normalised by AVP-AAV in both the SON and medial amygdala (MeA), but not in the central and basolateral amygdala or lateral hypothalamus. In vitro electrophysiological recordings showed an AVP-induced inhibition of MeA neurons that was prevented by picrotoxin administration, supporting the possible regulatory role of AVP originating in the SON. A memory deficit in the novel object recognition test seen in di/di animals remained unaffected by AVP-AAV treatment. Interestingly, although di/di rats show intact social investigation and aggression, the SON AVP-AAV treatment resulted in an alteration of these social behaviours. AVP released from the magnocellular SON neurons may stimulate adrenocorticotropin secretion in response to defined stressors and might participate in the fine-tuning of social behaviour with a possible contribution from the MeA.

## 1. Introduction

The hypothalamic-pituitary-adrenocortical (HPA) axis is the major endocrine system involved in stress adaptation of the mammalian organism [1]. Historically, nonapeptide vasopressin (AVP) was the first molecule of hypothalamic origin for which a regulatory impact on HPA axis activity was reported (for review see: [2,3]). The isolation and characterization of the corticotropin-releasing hormone (CRH) in the early 1980s put the function of AVP as the regulator of HPA axis activity into a new perspective [4]. Subsequent studies revealed that in adult animals, AVP of hypothalamic origin, co-synthesized with CRH in the parvocellular neurons of the paraventricular nucleus of the hypothalamus (PVN), reaches the anterior pituitary through the long portal vessels and acts synergistically with CRH on local adrenocorticotropin (ACTH) synthesis and release [3]. It is of note that, in the mammalian brain, the vast majority of AVP is synthesized in magnocellular hypothalamic neurons located both in the PVN and in the supraoptic nucleus (SON). Although several authors suggested mechanisms by which AVP of magnocellular origin might contribute to HPA axis regulation [5,6,7], previous approaches to test this hypothesis by employing different manipulations (elimination or rescue of magnocellular AVP synthesis and application of defined stressors) and analysis techniques (c-Fos immunohistochemistry, electrophysiology, microdialysis, etc.) failed to provide conclusive results [2,5].

The use of animals from the spontaneous AVP-deficient Brattleboro strain (natural knockout, di/di) provides an alternative approach to investigate the contribution of magnocellular AVP to the control of HPA axis activity [8,9,10]. Previous studies in adult male di/di rats suggested that both immune challenge by intravenous (IV) egg white and hypoglycaemia-inducing insulin injections (as a metabolic stressor) resulted in reduced ACTH levels compared to respective controls [11]. In terms of stress-related behavioural alterations (anxiety), the results were not unequivocal, with lower anxiety levels in di/di animals compared to controls in some cases [12,13] but not in others [8,14,15]. However, AVP-deficiency resulted in reduced performance in many defined learning and memory tests [16,17], in agreement with the supposed role that AVP plays in the process [18,19,20,21]. Furthermore, AVP might influence social development [22] and play a crucial role in social behaviour neural network [23,24].

The administration of adeno-associated viral vectors (AAV) allows the long-term restitution of naturally occurring or engineered mutations. Previous studies, focusing on the suitability of this treatment, confirmed the normalisation of the increased water-intake of di/di rats by intra-SON injections of an AVP-AAV construct [25,26]. It is of note that the rescue of the AVP synthesis and its physiological relevance was detectable for more than one year, indicating a long-lasting restoration [25].

**Figure 1:**
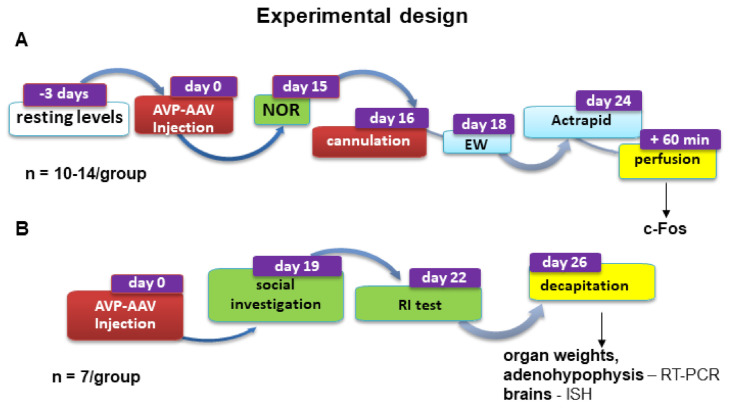
A schematic drawing illustrating the experimental protocol for the two separate series of male Brattleboro rats treated with bilateral AVP-AAV in the SON. (**A**) Series 1 and (**B**) Series 2. Colour code for the manipulations is as follows: blue, stressors and endocrine measures (ACTH and corticosterone); red, surgical procedures/treatments; green, behavioural testing; yellow, preparation for tissue collection. Abbreviations: ACTH, adrenocorticotropin; AVP-AAV, vasopressin-containing adeno-associated virus vector; EW, egg white injection; ISH, in situ hybridization; NOR, novel object recognition; RT-PCR, polymerase chain reaction; RI, resident intruder test; SON, supraoptic nucleus. For more details, see the text in Material & Methods.

The aim of the present study was to investigate the role magnocellular AVP plays in endocrine and behavioural regulation by restoring AVP synthesis within the SON of di/di Brattleboro rats using an AVP-AAV construct (referred to later as AVP-AAV treatment and the animals as di/di-AVP). First, the impact on endocrine stress regulation was studied. Resting mRNA levels relevant for signalling within the HPA axis were examined by RT-PCR and in situ hybridization with the aim of seeing whether AVP-AAV treatment could normalise the AVP-deficiency-induced chronic changes. In a subsequent experiment, acute stressor-induced blood stress hormone levels were evaluated. Second, to identify possible target areas of the magnocellular SON neurons, we mapped activated brain areas after insulin injection as a metabolic stressor. We hypothesized that the reduced c-Fos activation expected in di/di animals would be restored in di/di-AVP animals in areas directly influenced by AVP originating in the SON. As the c-Fos data suggested the involvement of the medial amygdala (MeA), we conducted in vitro electrophysiology on acute brain slices to support the notion that extracellular AVP may indeed influence the MeA neurons. Furthermore, the behavioural relevance of the SON AVP synthesis rescue was studied. At first sight, anxiety might be linked to stress and, thus, might have been a good choice. However, based upon our previous unclear data on di/di animals (see earlier), we did not concentrate on this behaviour. Rather, we focused on memory using the novel object recognition (NOR) test, as we repeatedly found a memory decline in di/di animals in this test (see earlier). Subsequently, based on the implication of the MeA and its role in the control of social interactions [27], we also evaluated the effect of AVP synthesis rescue on social behaviour, including aggression (for the experimental flow see Figure 1).

## 2. Results

In accordance with previous results [25,26], AVP-AAV injection into the SON restored the local AVP synthesis both morphologically (Supplementary Figure S1B) and functionally (Supplementary Figure S1A, Supplementary Table S1).

### 2.1. Resting HPA Axis Levels: Restoration of Adenohypophyseal Corticotropin-Releasing Hormone Receptor 1 Elevation by AVP Synthesis Rescue in SON

**Figure 2:**
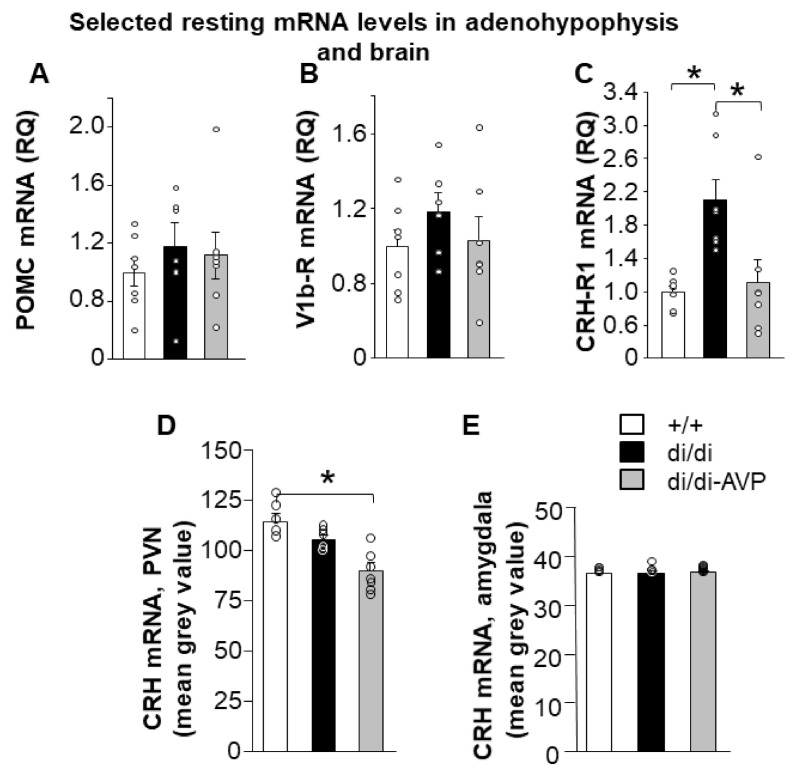
Levels of mRNA (individual values = circles and means + SEM) coding for defined peptides and proteins in the anterior pituitary (**A**–**C**), PVN (**D**) and amygdala (**E**) in Brattleboro rats after AVP synthesis rescue. As measured by RT-PCR, the mRNA levels of (**A**) POMC and (**B**) V1b receptor in the anterior lobe were not different between the groups. (**C**) At the level of the anterior lobe, the CRH-R1 mRNA level was found to be significantly increased in di/di rats compared to both other groups. In the PVN, the mRNA level of CRH measured by in situ hybridisation was decreased in di/di-AVP rats compared to +/+ (**D**), while no significant alterations were detected in the amygdala (**E**) (*n* = 7/group). Abbreviations: AVP, vasopressin; di/di, vasopressin-deficient Brattleboro rat; CRH, corticotropin-releasing hormone; CRH-R1, corticotropin-releasing hormone receptor 1; di/di-AVP, di/di animals with vasopressin synthesis rescue in the supraoptic nucleus; POMC, proopiomelanocortin; PVN, paraventricular nucleus of the hypothalamus; RQ, relative quotient; V1b-R, vasopressin 1b receptor. * *p* < 0.05, one-way ANOVA followed by Newman–Keuls comparisons.

Hypophyseal mRNA levels linked to HPA axis activity were measured by RT-PCR in the anterior lobe (Series 2). Both the proopiomelanocortin (POMC) and vasopressin 1b receptor (V1b-R) mRNA levels did not differ between the groups (Figure 2A,B). However, corticotropin-releasing hormone receptor 1 (CRH-R1) mRNA was enhanced in di/di compared to +/+, whereas di/di-AVP animals did not differ from +/+ (Figure 2C; F(2,18) = 7.993, *p* = 0.003). The CRH-R1 mRNA level correlated positively with water consumption at day 14 (*r* = 0.749, *p* < 0.0001).

Hypothalamic CRH mRNA levels were measured by in situ hybridization (Series 2) with significant differences between the groups detected in the PVN (Figure 2D; F(2,18) = 14.109, *p* = 0.0004), but not in the amygdala (subregions were not separated, our samples contained mainly the central amygdala (CeA) [28]) (Figure 2E). Namely, CRH mRNA in the PVN tended to be lower in di/di compared to +/+ (*p* = 0.096), with significantly lower levels in the di/di-AVP group compared both to +/+ and di/di rats (*p* = 0.0007 and *p* = 0.006, respectively). The CRH mRNA level in the PVN correlated negatively with the body weight change (*r* = −0.494, *p* = 0.044).

**Figure 3:**
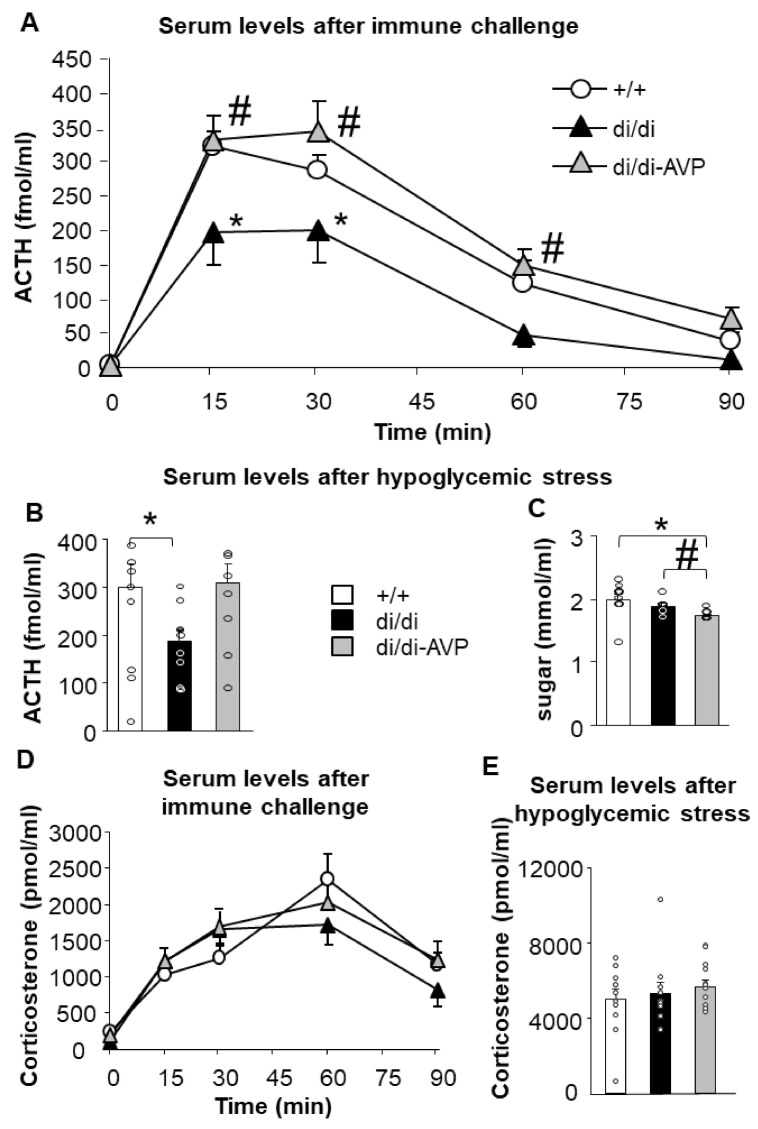
Blood serum ACTH and corticosterone concentrations (individual values = circles and means ± SEM) in response to acute stressors in Brattleboro rats after AVP synthesis rescue. Immune challenge by intravenous egg white injection significantly elevated the (**A**) ACTH and (**D**) corticosterone levels with a blunted ACTH, but not corticosterone response in di/di rats; di/di-AVP animals responded with ACTH levels similar to +/+ (*n* = 8–10/group). (**B**) Actrapid (fast-acting insulin) treatment-induced ACTH rise just failed to reach significance in terms of a smaller response in di/di (*p* = 0.05), but not in di/di-AVP rats. (**E**) We found no differences between the groups in corticosterone levels (*n* = 8–10/group). (**C**) Blood glucose levels were significantly lower in di/di-AVP animals compared both to +/+ and di/di (*n* = 8–10/group). Abbreviations: ACTH, adrenocorticotropin; AVP, vasopressin; di/di, vasopressin- deficient Brattleboro rat; di/di-AVP, di/di animals with vasopressin synthesis rescue in the supraoptic nucleus. * *p* < 0.05 significant difference from +/+ at the same time point, # *p* < 0.05 significant difference from di/di at the same time point, two-way (**A**,**D**) or one-way (**B**,**C**,**E**) ANOVA followed by Newman–Keuls comparisons.

Resting serum ACTH and corticosterone concentrations failed to differ between all groups in Series 1 (jugular venous catheter at min 0; Figure 3, individual values: Supplementary Table S2) and in Series 2 (trunk blood; data not shown).

### 2.2. Restoration of Acute Stress-Induced Lower ACTH Levels in AVP-AAV-Treated Brattleboro Rats (Series 1)

A immune challenge by IV egg white injection enhanced the serum ACTH concentrations (time: F(4,88) = 107.500, *p* < 0.0001), but the effect was different in the three groups (Figure 3A; group: F(2,22) = 7.507, *p* = 0.003; group × time: F(8,88) = 2.075, *p* = 0.047). Di/di animals showed lower ACTH values than +/+ at 15 and 30 min after the IV immune challenge, while AVP synthesis rescue in the SON normalised these levels. The same stimulus was also able to increase the serum corticosterone levels (time: F(4,104) = 46.950, *p* < 0.0001), without causing significant effects on the factors “group” or “time × group interaction” (Figure 3D).

Metabolic stress resulted in a tendency for group differences (F(2,27) = 2.874, *p* = 0.074), just failing to reach significance for lower ACTH levels in di/di compared to +/+ (*p* = 0.051) (Figure 3B). The di/di-AVP groups had similar levels as the +/+ animals (*p* = 0.868), and there was a tendency for a difference between di/di and di/di-AVP animals (*p* = 0.087). Sixty min after the injection of insulin, the corticosterone levels were similar in all groups (Figure 3E).

To monitor the physical impact of the stimulus application, we measured the blood glucose levels (Figure 3C). Interestingly, there was a significant difference between the groups 60 min after insulin injection (F(2,29) = 3.874, *p* = 0.032) with lower levels in the di/di-AVP group compared to both the +/+ and di/di groups (*p* < 0.028 and *p* = 0.040, respectively). The blood sugar levels did not correlate with the ACTH or corticosterone levels; thus, the subjective severity of the stressor cannot easily explain the observed hormonal changes presented above.

### 2.3. Altered Hypoglycaemic Stress-Induced c-Fos Signal in the SON and MeA of di/di Rats Is “Normalised” by AVP-AAV Treatment (Series 1)

**Figure 4:**
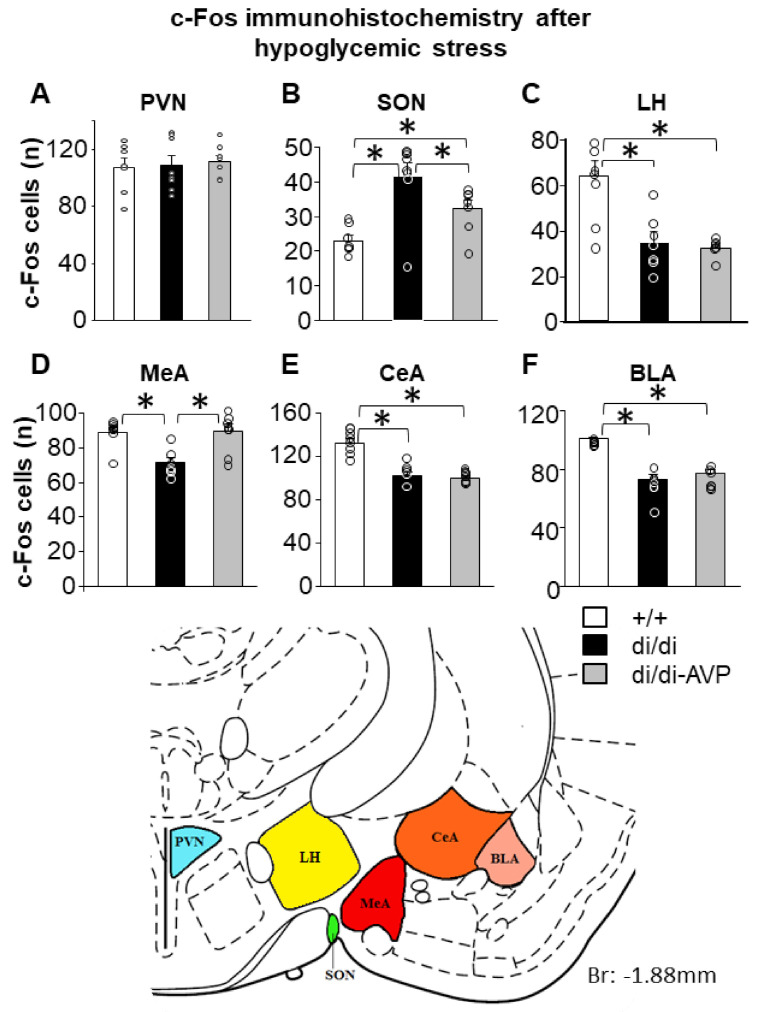
The number of c-Fos immunoreactive cells (individual values = circles and means + SEM) counted in defined brain areas 60 min after insulin injection in male Brattleboro rats after AVP synthesis rescue. Unlike in the PVN (**A**) di/di animals showed a higher number of c-Fos positive cells in the SON (**B**) and a reduced number in LH, (**C**) MeA (**D**), CeA (**E**) and BLA (**F**) than +/+. Please note that in di/di-AVP animals, this difference was normalized in the SON and MeA only. The schematic drawing obtained from a rat brain atlas illustrates the reference brain areas selected for the microscopic analysis (*n* = 7–8/group). Abbreviations: AVP, vasopressin; BLA, basolateral amygdala; CeA, central amygdala; di/di, vasopressin-deficient Brattleboro rat; di/di-AVP, di/di animals with vasopressin synthesis rescue in the supraoptic nucleus; LH, lateral hypothalamus; MeA, medial amygdala; PVN, paraventricular nucleus of the hypothalamus. * *p* < 0.05, one-way ANOVA followed by Newman–Keuls comparisons.

Insulin injection failed to differently affect the number of c-Fos positive cells within the PVN (Figure 4A). In contrast, in the SON a higher number of c-Fos positive cells that were recorded in di/di compared to +/+ animals (Figure 4B, F(2,19) = 9.915, *p* = 0.001). The number of c-Fos positive cells in +/+ was similar to that seen in di/di-AVP rats. In the lateral hypothalamus (LH; located dorsally to the SON and laterally to the MeA) both the di/di and di/di-AVP animals showed a lower c-Fos response to hypoglycaemia when compared to the +/+ (Figure 4C, F(2,19) = 12.721, *p* < 0.0001). Similarly, we counted a reduced number of c-Fos positive cells in di/di rats within three sub-areas of the amygdala compared to +/+ animals (Figure 4D, MeA: F(2,21) = 10.803, *p* = 0.001; Figure 4E, CeA: F(2,21) = 37.782, *p* < 0.0001; Figure 4F, basolateral amygdala (BLA): F(2,20) = 41.185, *p* < 0.0001). Interestingly, only in the MeA, but not in the CeA and BLA (also not in LH), the AVP-AAV treatment caused an increase in the number of c-Fos positive cells, reaching a level comparable to that of +/+ (for MeA di/di vs. +/+ and di/di vs. di/di-AVP: *p* = 0.001, +/+ vs. di/di-AVP: *p* = 0.883), thereby suggesting a significant impact of AVP with SON origin on the MeA.

The number of c-Fos positive cells in the MeA was positively correlated with the ACTH levels measured 15 min after egg white injection (*r* = 0.544, *p* = 0.029), suggesting that an insulin-induced hormonal change pointed in the same direction as changes in the neuronal activity in the MeA.

### 2.4. AVP Inhibited MeA Neurons through Involvement of GABA_A_ Receptors-Electrophysiological Recordings from Brain Slides

**Figure 5:**
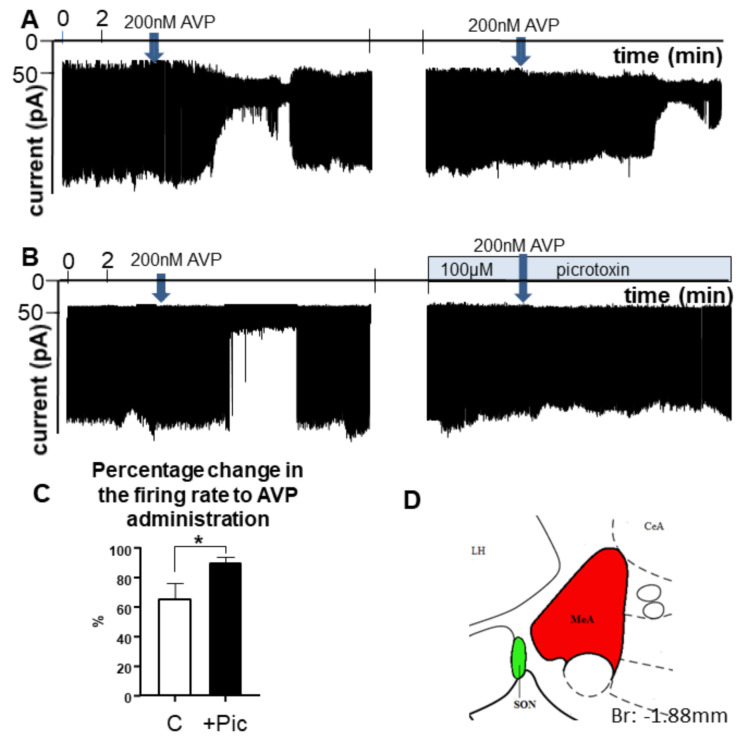
Representative examples of electrophysiological recordings illustrating the effect of AVP on the firing of MeA neurons in a brain slice obtained from +/+ rats. (**A**) Approx. 1–2 min after administration of 200 nM synthetic, AVP firing of MeA neurons was reduced. Repeated application of AVP resulted in a similar decrease. (**B**) The effect of AVP could be eliminated by the GABA_A_-R blocker picrotoxin. (**C**) The bar graph summarizes the results. The arrow indicates the time of application of the single bolus of AVP. (**D**) The inset shows the area of the slice that was chosen for the recording (*n* = 10). * = *p* < 0.05, Student’s *t*-test. Abbreviations: AVP, vasopressin, C: control, only AVP was administrated; MeA, medial amygdala; Pic, picrotoxin.

Loose-patch recording showed that AVP (200 nM) inhibited the spontaneous firing of MeA neurons, as represented in Figure 5. A decrease in the firing rate could be observed in 75% of the neurons recorded (66.07 ± 10.56% of the control value; t(9) = 9.0, *p* = 0.010; where the control level was 4.04 ± 0.805 Hz) (Figure 5A). The cessation of firing started within 1.45 ± 0.23 min of AVP administration. A washout could be observed after 8.32 ± 0.9 min when firing started to return. After recovery, a second recording started in the same neuron, showing that a second application of AVP resulted in a similar decrease in the firing rate.

In order to study the role of GABA_A_-R in the observed effect, AVP was applied to another group of neurons in the MeA (Figure 5B). After the first application of AVP, picrotoxin was added to the aCSF and, in the presence of this GABA_A_-R inhibitor, AVP was added again to the same neuron. The recording shows that picrotoxin significantly eliminated the action of AVP (90.1 ± 3.96%, t(18) = 18.0, *p* = 0.047). The bar graph summarizes the results: that AVP decreased the firing rate through the involvement of GABA_A_-Rs (Figure 5C).

### 2.5. AVP-AAV Rescue in the SON Had No Effect on NOR but Altered the Social Behaviour (Series 1 and 2)

**Figure 6:**
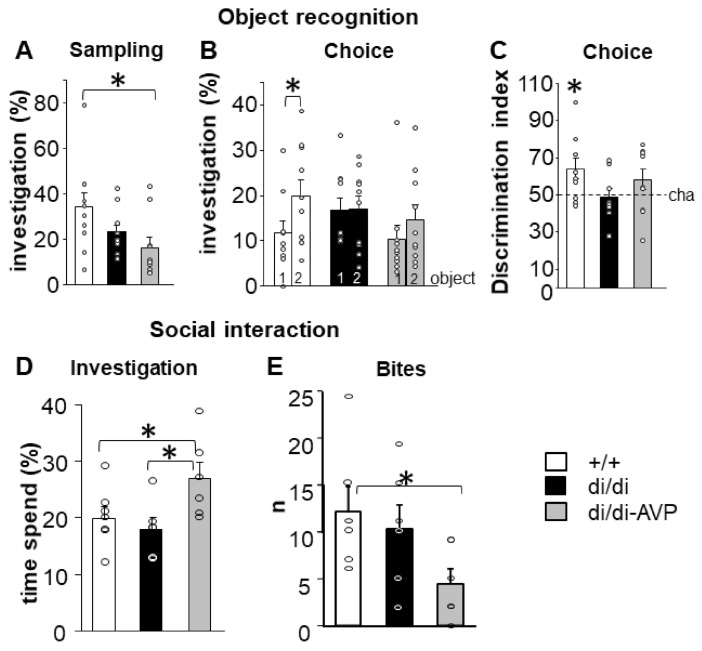
Behavioural parameters (individual values = circles and means + SEM) were measured in Brattleboro rats after AVP synthesis rescue. Object recognition (**A**–**C**): (**A**) during sampling di/di-AVP animals spent less time investigating object 1 (tested later as familiar) than +/+; (**B**) 30 min later, only +/+ rats were able to discriminate between old and new object by showing a significantly increased investigation duration towards the new object 2. (**C**) This is confirmed by the analysis of the discrimination index, where only +/+ animals show an index significantly higher than the chance level (=50%). Social interaction (**D**,**E**): (**D**) di/di-AVP animals investigated a previously not encountered conspecific juvenile significantly longer than both +/+ (*p* < 0.05) and di/di rats (*p* < 0.05). (**E**) In the resident-intruder paradigm, di/di-AVP animals showed a lower attack frequency than +/+ (*p* < 0.05), with a tendency to be lower than that of di/di animals (*p* = 0.07; *n* = 6/group). Abbreviations: AVP, vasopressin; di/di, vasopressin-deficient Brattleboro rat; di/di-AVP, di/di animals with vasopressin synthesis rescue in the supraoptic nucleus. * *p* < 0.05 Discrimination index: single sample *t*-test, Bites: Kruskal–Wallis test followed by Mann–Whitney post hoc comparison, Others: one-way ANOVA followed by Newman–Keuls comparisons.

During the sampling phase of NOR, the di/di-AVP animals spent less time investigating the novel object than the +/+ rats (Figure 6A, F(2,27) = 3.462, *p* = 0.046). However, even this lower level (40–55 s) should have been enough to remember the object [29]. During the choice phase, a memory deficit of the di/di rats was revealed by the NOR test (Figure 6B, difference between old and new objects: +/+: *p* = 0.047, di/di: *p* = 0.954, di/di-AVP: *p* = 0.224). The discrimination index also showed memory decline in di/di animals (Figure 6C, single sample *t*-test: +/+: *p* = 0.030, di/di: *p* = 0.808, di/di-AVP: *p* = 0.166). The AVP-AAV rescue failed to significantly affect the impaired memory performance.

During the social investigation test, the di/di-AVP rats spent more time investigating the conspecific juvenile than either the +/+ or the di/di animals (Figure 6D; F(2,16) = 3.938, *p* = 0.041).

In the RI test, the quantitative measure of aggression (number of bites) was reduced in the di/di-AVP rats compared to the +/+ (*p* = 0.030), also showing a tendency to be lower in comparison with the di/di group (*p* = 0.075) (Figure 6E; F(2,18) = 5.923, *p* = 0.051).

## 3. Discussion

Rescuing AVP synthesis in the magnocellular neurons of the SON resulted in the “normalisation” of ACTH secretion in the AVP-deficient Brattleboro rats in response to immune stimulus with a trend after a metabolic challenge (Figure 3). This was in parallel with a “normalisation” of the elevated resting CRH-R1 mRNA levels in the anterior pituitary, whereas other parameters linked to HPA axis activity under resting conditions were not affected by AVP deficiency, and therefore were also not rescued (CRH mRNA in the PVN, POMC, and V1b receptor mRNA in the anterior pituitary, as well as corticosterone at the periphery) (Figure 2).

Interestingly, AVP synthesis rescue in the SON “normalised” hypoglycaemia-driven c-Fos elevation of AVP-deficient rats in the MeA only, but not in its close vicinity (i.e., CeA, BLA, and LH) (Figure 4).

It is of note that despite the fact that there was no difference between +/+ and di/di animals in both social investigatory and aggressive behaviour, AVP-AAV treated di/di animals investigated conspecific juveniles significantly longer and showed a lower level of aggression than +/+ rats (Figure 6D,E). This suggests that AVP, originating in the SON, may have signalled on MeA neurons to promote non-aggressive social interactions. In contrast, the object memory deficit of the di/di rats was not corrected by AVP-AAV treatment (Figure 6B,C). This confirms a rather old hypothesis of a specific contribution of intra-SON AVP to social behaviour [30].

### 3.1. AVP of SON Origin and Stress Regulation

#### 3.1.1. Resting Levels

In line with our previous observation, AVP does not contribute to the maintenance of ACTH or corticosterone levels under resting conditions [11]. However, its constant absence stimulated CRH-R1 mRNA expression in the anterior pituitary (Figure 2C). Hauger and co-workers [31] linked pharmacologically high serum AVP levels to a downregulation of CRH-R1 in the anterior pituitary. Thus, we hypothesize that the missing peripheral AVP feedback of di/di Brattleboro rats (directly or through an influence on water consumption, indicated by a positive correlation between water consumption and CRH-R1 mRNA) contributed to their elevated CRH-R1 mRNA levels. Indeed, rescuing AVP synthesis (and release) resulted in “normalisation” of the elevated CRH-R1 mRNA levels in the present study. This suggests that AVP of magnocellular origin may contribute to an appropriate HPA axis response by fine-tuning the signal intensity of CRH on the corticotroph cells.

However, at the level of the PVN, the energy balance of the body seems to be the main determinant of the CRH mRNA content (see the negative correlation between CRH mRNA and body weight change), most probably through changes in water and (subsequently) food intake. Indeed, as CRH is an anorectic peptide [32], it seems plausible that its level should be reduced in di/di-AVP animals to be able to catch up with the body weight of +/+ rats under settled water uptake conditions. Further studies may analyze the plasma levels of AVP of the different treatment groups to obtain a more detailed insight into its impact on the release of the hormone after local rescue of its synthesis.

#### 3.1.2. Acute Stress

The contribution of magnocellular AVP to acute stress regulation was previously confirmed after osmotic challenges (including IP injections or drinking of hypertonic saline and water deprivation) by elevation of both the mRNA [33] and peptide levels [34,35], not only in the PVN, but also in the SON, consistent with the importance of AVP for the body’s salt-water homeostasis. For non-osmotic stressors, the following picture varies significantly: forced swimming [36] or immune challenge [37], for instance, failed to affect AVP mRNA in the SON, whereas water immersion restraint increased the number of c-Fos synthesizing AVP positive cells in the SON and PVN [38].

It has to be noted that changes in mRNA and protein levels of neuropeptides in the SON cannot easily be attributed to the central and/or peripheral release profiles [39]. Studies analysing the presence of AVP in the hypophyseal portal blood (which is thought to reach the anterior lobe and trigger ACTH release [40,41]) revealed that AVP remained detectable after lesioning the PVN, thereby suggesting its magnocellular and, thus, SON origin [42]. Moreover, direct stimulation of the SON, either by microdialysis with hyperosmotic solution [43] or electrically [44], triggered an elevation of the stress hormone levels. The fact that in the latter study, the corticosterone elevation was absent in rats with lesioned PVN [44] may relate to the elimination of the permissive action of CRH on AVP-mediated ACTH secretion [6]. Nevertheless, these data were indirect measures and/or collected under unstressed conditions.

One goal of the present study was to test the hypothesis that AVP of magnocellular origin acts as an ACTH-releasing factor [45] during defined exposure to a stressor. We have chosen egg whites as an immune challenge and insulin-induced hypoglycaemia as a metabolic stressor. We have repeatedly reported that in AVP-deficient Brattleboro rats, ACTH response is decreased in response to both stimuli [11,46,47]. However, in those studies, AVP was absent from both the parvo-and magnocellular neurons of the di/di animals. The data obtained here support the hypothesis of a direct contribution by AVP of magnocellular origin to acute HPA axis regulation (Figure 3). As a single immune challenge may induce long-lasting changes in the HPA axis sensitivity [48], we cannot entirely exclude the possibility that the data collected after insulin injection might have been contaminated by the effects of egg white applied 6 days before. This could explain why, in the latter case, there were only tendencies between the groups.

### 3.2. Possible Brain Targets of AVP Originating in the Magnocellular SON

#### 3.2.1. c-Fos Data

The lack of any difference between the groups in the PVN together with the changes seen in SON confirmed that our vector treatment affected only the latter (see also Supplementary Figure S1B).

Previous studies reported decreased c-Fos activation in the MeA and nucleus accumbens of the di/di mothers [14], as well as in the CeA, PVN, lateral septum, and nucleus arcuatus of the di/di females [49] in response to a 15 min forced swimming session, when compared to the respective controls. Similarly, the increase in the number of c-Fos positive cells after an acute IP injection of hypertonic saline was significantly less pronounced in the SON of the male di/di animals [50]. In this context, it is of note that, under basal conditions, the c-Fos immunoreactivity was reported to be higher in the SON of the di/di compared to the +/+ animals [51]. Here, we showed that insulin-injection induced lower neuronal activation in MeA, BLA, CeA, and LH of di/di animals. We cannot entirely rule out the possibility that the chronic osmotic overload of the di/di rats contributed to the reduced c-Fos levels. Nevertheless, the lower neuronal activation in several brain areas went in parallel with the smaller ACTH response of the di/di rats, suggesting an involvement of the defined limbic brain areas in the generation of the endocrine response. This indicates that the latter is at least partially controlled by the emotional evaluation of the stressor. In this context, the MeA seemed to be particularly interesting. Indeed, other authors have shown that lesioning the MeA significantly reduces the conditioned fear-induced ACTH response [52].

#### 3.2.2. MeA and Vasopressin

We propose that the normalisation of c-Fos in the MeA of the di/di-AVP rats reflects the persistent influence of AVP originating in the SON rather than a specific response to hypoglycaemic stress. AVP of SON origin might have influenced the MeA neurons, reaching this area either by “volume transmission” via the extracellular space [53,54,55] or through direct innervation (confirmed in CD1 mice [56]). In female rats of the Sprague–Dawley strain, there is some indication that oxytocinergic SON neurons have sparse collaterals that also target the MeA [57]. Nevertheless, AVP receptors are present in the MeA (mostly V1a receptors [58,59,60,61,62], but the presence of V1b receptors cannot be entirely excluded [63,64,65]).

As MeA neurons are able to synthetize AVP [8], and the area is relatively close to the SON, we cannot entirely rule out the possibility that sparse MeA neurons might have been infected by the rAAV construct. However, it is doubtful that the few possible cells would be responsible for the full restoration of the neuronal signalling in this area.

#### 3.2.3. In Vitro AVP Sensitivity of MeA Neurons

During a previous study, in the MeA, 5 out of 28 cells (18%) were excited by the application of AVP at 300 nM, while 23 cells (82%) remained unresponsive [66]. In contrast, we found only an inhibitory effect of 200 nM AVP in all spontaneously firing cells (Figure 5). The discrepancy between previous results and ours may be explained by the different methodologies (e.g., recording spontaneous firing vs. silent cells). An identical result to that presented here was observed in an independent experiment, in which brain slices from Wistar rats were used in our laboratory under otherwise similar conditions (data not shown). This suggests that the AVP action in the MeA reported here can be observed in other rat strains in a reproducible manner.

The AVP effect in the MeA was significantly reduced by a pre-treatment with a GABA_A_-R antagonist, suggesting that AVP exerts its inhibitory action via interaction with local GABA signalling. The rodent’s MeA contains numerous GABAergic neurons [67,68]. They may provide the morphological substrate not only for the local GABA_A_-R antagonist action but also for the observed behavioural alteration, as they are known to be involved in the processing and generation of chemosensation [69], and aggressive behaviour [70,71].

### 3.3. Behavioural Influence of AVP Originating in the Magnocellular SON

#### 3.3.1. Effect on Object Recognition Memory

Because of the suggested beneficial impact of endogenous AVP on different types of memory [21,72,73], we examined whether AVP-AAV rescue in the SON could normalise the recognition memory performance deficit of di/di animals [16,17]. Our findings imply that the disturbed short-term memory of the di/di rats in the NOR test could not be restored by rescuing the AVP synthesis in the SON (Figure 6A–C). This is in line with the shortcomings of the AVP-memory hypothesis [74]. More precisely, in di/di animals the absence of AVP in the olfactory [73] and retinal system [75], as well its relevance for blood pressure regulation [74], might confound the results in memory tests and cannot be easily restored by hypothalamic AVP synthesis rescue. Previous studies suggested that intact AVP signalling in the rodent septum is essential for “normal” performance in recognition memory [19]. This septal information processing does not seem to be affected by our AVP-AAV treatment of the SON. We therefore focused subsequently on the relevance of AVP signalling in social behaviour [73,76].

#### 3.3.2. Effects on Social Behaviour

It was suggested that AVP may differently regulate social behaviour, acting on different brain regions [77]. For instance, higher AVP levels within the lateral septum stimulated aggressive behaviour, while within the bed nucleus of stria terminalis, it was inhibited [78]. A similar discrepancy was observed for the endogenous AVP release in the septum *versus* the amygdala for the control of coping behaviour during forced swimming [79,80]. This could explain how breaking the balance of extrahypothalamic AVP signalling in the brains of the di/di rats by rescuing AVP synthesis in the SON might have unmasked the role of this nucleus in social behaviour (Figure 6D,E). The change in social interactions was not the consequence of a general increase in investigatory curiosity, because during sampling in NOR, di/di-AVP animals showed an even lower level of object investigation than +/+ rats (Figure 6A). This implies that AVP signalling in the SON may selectively affect social curiosity and may contribute to fine-tuned social behaviour in concert with other neurotransmitters/neuromodulators. In this respect, AVP signalling in the MeA (suggested also by our data, see Section 4.2) might be especially important, as this area is sensitive to chemosensory signals, particularly non-volatile ones [81,82]. That might be the background for its involvement in social recognition [27]. Indeed, an MeA lesion in mice reduced aggressive behaviour either permanently or temporarily [70], and decreased social investigation [27]. Interestingly, intra-MeA (but not ICV) administration of a V1a antagonist significantly impaired maternal recognition (measured by maternal behaviour latencies) [83]. This supports not only the hypothesis of the importance of AVP signalling within the MeA via V1a receptors for social behaviour, but also our idea that the systemic manipulation of AVP signalling (i.e., by ICV injection or in a general knockout animal) may trigger both inhibitory and excitatory effects, compensating for each other and, therefore, resulting in unchanged behaviour. However, further studies have to evaluate the physiological impact of AVP originating in the SON on information processing in the MeA, since in normal non-=AVP-deficient animals, locally synthetized AVP might dominate the interaction with intra-MeA AVP receptors [8].

It is worth mentioning that the role of AVP might depend on the strain as well as on sex [49,84]. Moreover, the cellular morphology of the MeA is sexually dimorphic [85]. Thus, further studies should emphasize the sex differences.

## 4. Materials and Methods

### 4.1. Animals

Adult male Brattleboro rats (*n* = 71) from the breeding colony of the Institute of Experimental Medicine in Budapest, Hungary, were kept under controlled laboratory conditions (12 h light/12 h dark, with lights on at 7:00 a.m.). The animals had free access to food and tap water and were individually housed in Makrolon cages (40 cm × 25 cm × 25 cm) containing sawdust bedding (Charles River, Veszprém, Hungary) at the beginning of the experiments to control for individual water consumption. Cage bedding was changed daily; further details about the breeding and genotyping are given elsewhere [86]. Di/di and control (+/+) Brattleboro rats (8–10 months old at the beginning of the experiment) were selected according to their age, so that their body weight showed a difference of ~20% [86]. All experiments were done between 9:00 a.m. and 1:00 p.m., at the nadir of stress hormone levels [11]. Experiments performed with Brattleboro rats were conducted in two separate series. In both series half of the di/di animals were treated with AVP-AAV(di/di-AVP), while all other rats underwent a sham operation. The exact protocol for each series is described below and is illustrated in Figure 1.

Adolescent male +/+ rats (~35 days old; *n* = 3) were used for the electrophysiological experiments and Wistar juveniles (~25 days old; *n* = 16) as social stimuli in the social investigation test, with Wistar adults (~50 days old; *n* = 10) as intruders in resident-intruder test (see below). These rats were kept under housing conditions similar to those described above except for being group-housed (3–5 per cage) and with the bedding changed twice per week.

### 4.2. Stereotaxic AVP-AAV Injection

Anaesthesia was performed by an intraperitoneal (IP) injection of a mixture of ketamine (50 mg/kg, SelBruHa Állatgyógyászati Kft., Budapest, Hungary), xylazine (20 mg/kg, Spofa, Prague, Czech Republic) and promethazinium chloratum (0.2 mL/kg, Egis, Budapest, Hungary) dissolved in physiological saline [11].

Anaesthetised rats were fixed in a stereotaxic frame (David Kopf Instruments, Tujunga, CA, USA). To rescue local AVP synthesis in the Brattleboro rat, AVP-AAV (Type 2, CMV-GH-SP-AVP-NP-GP-SVPA, 7 × 10^9^ genome copies/µL, 100 nL/injection site; for more information about the vector see [25]) was bilaterally injected into the SON of di/di rats (stereotaxic coordinates from Bregma: AP: −0.4 mm, ML: +/−1.8 mm, DV: +9.7 mm) (later referred to as di/di-AVP) [8]. Controls (both +/+ and di/di animals) were injected with saline on the same day and in the same volume as no proper control virus was available. In contrast to Ideno et al. [25] in the present experiment we did not use the beta-galactosidase-containing control vector, as previous studies [87], as well as our preliminary examinations, showed that the galactosidase nucleotide sequence might have a significant impact on the behaviour. We cannot entirely exclude the possibility that the virus incorporation per se induced some unwanted effects in treated animals. However, at present all available data are against this possibility, since AAVs are unable to reproduce, they are non-pathogenic and do not incur an immune response [88].

Only di/di-AVP animals showing a significant drop in water consumption were included in subsequent experiments (10 out of 18 for Exp. 1 and 7 out of 10 for Exp. 2, see Supplementary Figure S1A), as in this strain water consumption clearly defines the correct hits [25,89]. Although the cytomegalovirus (CMV) promoter used in the applied AAV construct is not tissue-specific, the presence of prohormone convertase in the cell is essential to produce mature AVP (for details see [13]). Therefore, in our hands transgene-derived AVP was specifically produced in the neurons of the SON of di/di-AVP rats, and its surrounding tissue could not produce mature AVP peptide despite a possible leakage of the vector solution [25,26]. We considered the drop in water consumption as a proof for successful functional rescue of AVP synthesis in the SON and we therefore started to test the animals two weeks after treatment. The immunohistochemical data confirmed the rescue (in the SON of +/+ rats 23% of the cells showed AVP positivity, while in di/di-AVP animals 17.5%) (see Supplementary Figure S1B).

### 4.3. Experimental Design for Stress and Behavioural Experiments

#### 4.3.1. Series 1

Water consumption and body weight of the rats were measured (daily and weekly, respectively) during the whole experiment (see Supplementary Figure S1A and Table S1). Fourteen days after AVP-AAV injection, when the water consumption was stable and reduced to normal levels in AVP-AAV treated animals with correct hits, a test for novel object recognition (NOR) was conducted. On the 16th day, a catheter was implanted into the right jugular vein under anaesthesia (see earlier). Repeated blood samples were collected on the 18th day and each rat received an IV injection of egg white immediately after the collection of the first blood sample [11]. After additional six resting days (on the 24th day), after 18 h fasting all animals received a bolus of IP Actrapid (fast acting insulin). Sixty minutes thereafter the animals were slightly restrained and blood was taken from a tail cut within 1 min. The rats were then immediately deeply anaesthetised and transcardially perfused. Fixed brains were removed from the skulls and used for immunohistochemistry.

#### 4.3.2. Series 2

The previous experiments revealed that the MeA, a nucleus known, among others, for its involvement in the control of social behaviour in rodents [27], responded to our rescue of AVP synthesis in the SON (see below). We therefore analysed the impact of AVP-AAV SON treatment on social behaviour. Eighteen days after AVP-AAV injection the animals were tested for their social investigatory behaviour and three days later for aggression. After four additional days of rest in the home cage rats were decapitated under resting conditions, the pituitary and brain were harvested, and blood was collected on water-ice. The serum was stored for the measurement of hormone concentrations by radioimmunoassay. The brain and anterior pituitary were frozen on dry ice and stored for further analyses.

### 4.4. Immunohistochemistry

In Series 1, 60 min after insulin injection and immediately after blood sampling from tail cuts rats were deeply anaesthetised. Then they were transcardially perfused with 100 mL of chilled phosphate-buffered saline (PBS; pH 7.4) followed by ice-cold 300 mL 4% paraformaldehyde (PFA, Molar Chemicals Kft., Halásztelek, Hungary) solution. After overnight post-fixation brains were transferred into a cryoprotective 30% sucrose solution in PBS for 2 nights at 4 °C and then stored at −80 °C until sectioning. Sequential 30-µm-thick coronal sections of the hypothalamus containing the SON and PVN were cut with a sliding microtome and divided into 6 parallel slice series. Supplementary Table S3 summarizes the protocol used.

#### 4.4.1. AVP Immunohistochemistry

Brain slices containing the SON and PVN were selected and stained for AVP. The AVP antibody was generated by Tamás Görcs [90,91]. Secondary antibodies were purchased from Jackson ImmunoResearch, Ely, UK. Cell nuclei in the slices were visualized by Hoechst 33258 (1:20,000; Sigma-Aldrich, Darmstadt, Germany). Slides were covered by Mowiol (Sigma-Aldrich).

Images were taken by Nikon C2 confocal laser scanning microscope in the Nikon Microscopy Centre at the Institute of Experimental Medicine in Budapest.

#### 4.4.2. c-Fos

The c-Fos antibody (sc-52) was purchased from Santa Cruz Biotechnology, Dallas, TX, USA, and the secondary antibody from Jackson ImmunoResearch, Ely, UK [49]. The avidin–biotin complex (ABC) was obtained from Vector Laboratories, USA, and the peroxidase reaction was developed in the presence of 3,3′-diaminobenzidine tetrahydrochloride (Sigma-Aldrich) (0.2 mg/mL), nickel–ammonium sulphate (0.1%) and H_2_O_2_ (0.003%). Slices were mounted on glass slides in gelatine solution (0.5% gelatine, 0.05% Chromium (III) sulphate; Sigma-Aldrich), then dehydrated with xylol isomer mixture and covered by DPX (Sigma-Aldrich).

Section planes were standardized according to the atlas of Paxinos and Watson [92]. Images were taken with a digital camera coupled to a bright-field microscope (Olympus BX51), with no further modifications. To select c-Fos immunopositive nuclei as targets for quantification, PC-based software (Scion Image, Scion Corporation, Frederick, MD, USA) was used and targets were subsequently identified in the captured images by grey level thresholding. Size criteria (minimum area of 20 pixels at a magnification ×10) were applied to exclude structures other than c-Fos immunopositive nuclei from measurement. The average of four sections taken at 150 μm intervals was used for the analysis of each structure, including both hemispheres; that is, 8 measures/area/animal.

The following brain areas were analysed (Figure 4): PVN, SON, LH, MeA, CeA, and BLA. First, we focused on brain areas that might be involved in HPA axis regulation (SON and PVN). Next, the brain areas located near the SON seemed to be a logical choice (either because of the supposed diffusion of AVP or the presence of short axonal collaterals [93]). Certainly, we cannot rule out the possibility that other, more remote brain areas might also have been influenced.

### 4.5. Quantitative Real-Time PCR Measurements

Animals used in Series 2 were decapitated under resting conditions, the pituitaries were quickly removed from the skull, the anterior lobes were dissected under a microscope and placed in an RNase-free plastic tube and frozen on dry ice until further processing.

Each frozen anterior pituitary was homogenized in 500 μL QIazol Lysis Reagent (QIAGEN, Valencia, CA, USA) and the total RNA was isolated with QIAGEN RNeasy MiniKit (QIAGEN, Valencia, CA, USA) according to the manufacturer’s instructions. Sample quality control and quantitative analysis were carried out by NanoDrop (Thermo Fisher Scientific, Waltham, MA, USA). cDNA synthesis was performed with the High-Capacity cDNA Reverse Transcription Kit (Applied Biosystems, Foster City, CA, USA). The primers (Invitrogen, Waltham, MA, USA) were used in a real-time PCR (RT-PCR) with a Power SYBR Green PCR master mix (Applied Biosystems, Foster City, CA, USA) on an ABI StepOnePlus instrument (Applied Biosystems). The primer pairs were designed according to Supplementary Table S4.

Gene expression profiles were analysed using the ABI StepOne 2.3 program (Applied Biosystems). The amplicons were tested by melt curve analysis on an ABI StepOnePlus instrument (Applied Biosystems) [17]. Results were normalised to ribosomal protein S18 (RPS18) expression in the anterior pituitary [94]. Relative quantity of mRNAs was referred to corresponding samples of the +/+ animals based upon the 2^−^^ΔΔCT^ method (ABI StepOne 2.3 program, Applied Biosystems). The RNAs of the following molecules were analysed: POMC as the precursor of ACTH; V1b-R as the main AVP receptor in the anterior pituitary; CRH-R1 as the main CRH receptor in the anterior pituitary.

### 4.6. In-Situ Hybridization

Resting animals from Series 2 were decapitated, the brains were quickly removed and frozen in dry ice cold isopentane. In situ hybridization was performed as described earlier [95]. Briefly, 12 μm thick serial coronal sections of the hypothalamus were cut in a cryostat (Leica Microsystems GmbH, Wetzlar, Germany). The sections were thaw-mounted and air-dried at 37 °C onto positively charged Superfrost Plus slides (Thermo Scientific, Budapest, Hungary), and kept at −80 °C until use. A 498 bp-long fragment of the rat CRH cDNA (accession #: NM_031019; kindly provided by Young WS 3rd) was subcloned into a pBluescript II SK vector and used as template for in vitro transcription of [^35^S]UTP-labelled probes, according to the MAXIScript T7 Kit (Invitrogen). Hybridisations were performed overnight in humid chambers at 55 °C with 10^6^ cpm/slide of the [^35^S]UTP-labelled probes. After washing steps the slides were opposed to a BAS-MS imaging plate (Fuji Photo Film Co., Ltd., Kanagawa, Japan,) for 3 days, and data were then read out by a Fujifilm FLA-8000 Image Analyser. The signals from the areas of interest (PVN and amygdala) were standardized according to the atlas of Paxinos and Watson [92] and carefully matched between the animals. Optical densities (mean grey values) were evaluated on the phosphor imager recordings by using the ImageJ 1.32j software (Wayne Rasband, NIH, MD, USA, http://rsbweb.nih.gov/ij/ (accessed on 12 April 2016)) [96]. Measurements were performed on 3 sections for PVN and 7–10 sections for amygdala (including MeA, BLA, CeA) separately for the left and right sides using the same settings across animals. Background values were measured in parallel and were subtracted. The average/animal data were used for statistical evaluation.

### 4.7. Stress Experiments

#### 4.7.1. Immune Challenge

Two weeks after virus injection, the animals of Series 1 were anaesthetised as described above, the catheter was implanted into the right jugular vein and the animals were given two days to recover. On the day of the experiment, the catheter was connected to a ~50 cm long polyethylene tube and after collection of the first blood sample the rats were slowly injected with fresh, filtered egg white (500 mL/L solution in sterile saline) through the jugular catheter in a dose of 1 mL/kg [11]. Blood samples (0.4 mL/sample) were taken at 15, 30, 60, and 90 min subsequently from freely moving, unrestrained animals via the catheter. The volume of the samples was replaced by physiological saline to avoid a drop in circulating blood volume. At the termination of this experiment rats were infused with saline via the catheter and it was closed.

#### 4.7.2. Metabolic Stressor

Six days later and after 18 h fasting (Series 1), hypoglycaemia was induced by IP insulin injection (Actrapid, a fast-acting insulin, 3 IU/2 mL/kg; Novo Nordisk, Bagsværd, Denmark). One hour later blood was taken from a tail cut to measure ACTH, corticosterone and blood glucose [11]. Subsequently, the animals were anaesthetised and transcardially perfused for c-Fos immunohistochemistry. Blood glucose levels were measured with a commercially available analyser (D-Cont Personal, 77 Elektronika Kft, Budapest, Hungary).

### 4.8. Hormone Measurements

Blood samples were collected in ice-cold plastic tubes and centrifuged (30 min at 3000× *g*). The serum was separated and stored at −20 °C until analysis. ACTH and corticosterone were measured by radioimmunoassay in 50 μL and 10 μL of unextracted serum, respectively, as described earlier [97]. The intra-assay coefficients of variation for ACTH and corticosterone were 4.7% and 7.5%, respectively. All the samples from a given experiment were measured in the same radioimmunoassay.

### 4.9. Electrophysiological Recordings

AVP receptors (presumably V1a) can be found on vascular smooth muscle cells [98], via which AVP administration into brain tissue can change the local blood supply and influence the activity of a brain area inducing indirect electrophysiological responses [12]. To overcome the confounding vasoconstrictor effect we used brain slices, as in this case circulation is not an important component of tissue nutrition [99].

The concentration of AVP in the cerebrospinal fluid (CSF) collected from the cisterna magna of rats was reported to be of the order of 10^−2^ nM [100]. It is known that in the brain tissue extracellular AVP concentrations are magnitudes higher, and 200 nM AVP was previously shown to be able to influence electrical activity of CeA neurons [66].

Loose-patch measurements were carried out to record action currents in neurons of the MeA of the acute brain slice as described earlier [101]. Briefly, the rats were deeply anaesthetised using isoflurane inhalation. The brain was removed rapidly and immersed in ice cold sodium-free artificial CSF (Na-free aCSF) bubbled with a mixture of 95% O_2_ and 5% CO_2_. The solution contained the following substances (in mM): sucrose 205, KCl 2.5, NaHCO_3_ 26, MgCl_2_ 5, NaH_2_PO_4_ 1.25, CaCl_2_ 1, and glucose 10. Blocks from the brain were dissected and 250 μm-thick coronal slices were prepared from the area containing the MeA (Figure 5) with a Leica VT-1000S vibratome (Leica Microsystems, Wetzlar, Germany) in the ice-cold oxygenated Na-free aCSF. The slices were then equilibrated in the following normal aCSF (in mM): NaCl 130, KCl 3.5, NaHCO_3_ 26, MgSO_4_ 1.2, NaH_2_PO_4_ 1.25, CaCl_2_ 2.5, glucose 10, and saturated with O_2_/CO_2_ for 1 h. The initial temperature of aCSF was 33 °C and it was left to cool down to room temperature during equilibration.

Recordings were carried out in oxygenated aCSF at 33 °C. Axopatch 200B patch-clamp amplifier, Digidata 1322A data acquisition system, and pCLAMP 10.4 software (Molecular Devices Co., San Jose, CA, USA) were used for recording. Cells were visualized with a BX51WI Ir-Dic microscope (Olympus Co., Tokyo, Japan) located on a stable antivibration table (Supertech Co., Pecs, Hungary). Thxe patch electrodes (OD = 1.5 mm, thin wall, Hilgenberg GmBH, Malsfeld, Germany) were pulled with a Flaming/Brown P-97 puller (Sutter Instrument Co., Novato, CA, USA) and polished with an MF-830 microforge (Narishige Inc., Tokyo, Japan). Pipette potential was set to 0 mV, pipette resistance was 1–2 MΩ, resistance of loose-patch seal 7–40 MΩ. The pipette solution contained (in mM): NaCl 150, KCl 3.5, CaCl_2_ 2.5, MgCl_2_ 1.3, HEPES 10, and glucose 10 (pH = 7.3, adjusted with NaOH). The measurements started with an initial control recording (5 min), then (Arg^8^)-vasopressin trifluoroacetate salt (Bachem, Bubendorf, Switzerland; final concentration 200 nM, diluted in aCSF in the recording chamber [102]) was added to the aCSF in a single bolus onto the slice in the recording chamber and the recording was continued for a subsequent 10 min. In order to investigate the role of GABA_A_-R in the effect of AVP, we first examined whether repeated AVP administration would induce the same effect in the same cell. Next, after reproducing the AVP effect, picrotoxin (100 µM, Tocris, Budapest, Hungary; GABA_A_-R antagonist) was added to the bath fluid and was continuously present during rest of the recording including a second AVP administration.

Measurements were carried out on 10 neurons from 3 rats in each group. The percentage change in the firing rate was calculated by dividing the average frequency value during the 10 min period after AVP application with that of the 5 min period before AVP and multiplying by 100.

### 4.10. Behavioural Experiments

#### 4.10.1. Novel Object Recognition Test (NOR)

In Series 1, two weeks after AVP-AAV injection, rats were habituated to the experimental cages (41.3 × 26 × 29.8 cm^3^, Geo Maxi, Ferplast, Italy) for 60 min [17]. For recognition the following two different objects were used: a 62 g tin box and an 80 g glass bottle of tomato sauce. A defined object (Object 1) was presented for 4 min (sampling phase), then removed, and 30 min later the same (Object 1) together with a different object (Object 2) were introduced into the rat’s cage for another 4 min (choice phase). Objects were thoroughly cleaned with fresh tap water before each test. Tests were videotaped and subsequently analysed by an experimenter blind to the animals’ treatment using a computer-based event recorder (H77, Budapest, Hungary). Behaviour directed towards the object discriminated between sniffing and gnawing. To exclude object preference, the two objects were randomly used as Object 1 or Object 2, and to exclude place preference, the place of the presentation of Object 1 and Object 2 was also randomised. The discrimination index was calculated as follows: (time percentage Object 2)/(time percentage Object 1+ time percentage Object 2) × 100. A value of >50% is considered successful discrimination.

#### 4.10.2. Social Investigation

In Series 2, more than two weeks after AVP-AAV injection, the rats were transferred to a new cage (see at NOR) with fresh bedding 1 h before starting the test. The test consisted of a 4-min exposure to a previously not encountered conspecific juvenile (Wistar rat, ~25 days old). The duration of the investigatory behaviour of the adult towards the juvenile was measured online by a trained observer blind to the animal’s group, using an events recorder (EVENTLOG 1.0 written by Robert Hendersen 1986). Investigatory behaviour was defined as the direct action of the adult towards the juvenile rat including anogenital sniffing, licking, pawing, and close pursuit.

#### 4.10.3. Resident-Intruder Test (RI)

In Series 2, subjects were kept in the above-mentioned Geo Maxi cages for three days. The rats were then exposed in these (home) cages to smaller, unfamiliar Wistar opponents for 20 min. Their behaviour during the exposure was video recorded and scored later by an experimenter blind to treatment conditions. Behavioural analysis focused on the consummatory phase of aggressive behaviour i.e., on biting attacks [77]. The results of the quantitative measures (i.e., attack counts and latency) are presented, as we failed to detect differences in qualitative measures (i.e., attack type and context).

### 4.11. Statistics

Data are expressed as means ± SEM and analysed using the Statistica 13.0 software package (StatSoft, Inc., Tulsa, OK, USA). Data analysis was performed by analysis of variance (ANOVA) either using repeated measures (i.e., factors ‘groups’ and repeated factor ‘time’ for parameters water intake, and hormone levels measured after application of egg white as immune stressor) or one factor (factor ‘groups’). Post-hoc comparisons were made by the Newman–Keuls test. Electrophysiology data were analysed by student’s *t*-test. Discrimination index was evaluated by single-sample *t*-test against 50% chance level. In the case of aggressive encounters the frequency of bites was analysed by Kruskal–Wallis test followed by Mann–Whitney post hoc comparison. Correlations were calculated by the Pearson analysis. *p* values < 0.05 were considered significant, while between 0.05 and 0.1 were considered to indicate a trend.

## 5. Conclusions

Our data suggest that AVP synthesis and release originating in the magnocellular neurons of the SON might fine-tune the HPA axis response to acute stressors. Moreover, AVP originating from the SON might contribute, possibly via a pathway involving the MeA, to social behaviour. The present results corroborate our previous hypothesis that the modulation of neuronal functions by AVP plays an integrating role in social interactions [47]. AVP of SON origin may reduce aggression and increase social interest, thereby contribute to the fine-tuning of the rodent brain’s “social circuitry”.

## Data Availability

Data are available within the article and Supplementary Material. Raw data will be provided on request.

## Supplementary Materials

The following supporting information can be downloaded at: https://www.mdpi.com/article/10.3390/ijms23031357/s1.
