# Supplementary material for: Rescue of Vasopressin Synthesis in Magnocellular Neurons of the Supraoptic Nucleus Normalises Acute Stress-Induced Adrenocorticotropin Secretion and Unmasks an Effect on Social Behaviour in Male Vasopressin-Deficient Brattleboro Rats
Source: Int J Mol Sci. 2022 Jan 25;23(3):1357. doi: 10.3390/ijms23031357 (PMC8836014; doi:10.3390/ijms23031357)
Supplement: Supplementary file 1 [file ijms-23-01357-s001.zip › ijms-1540278-supplementary.pdf]

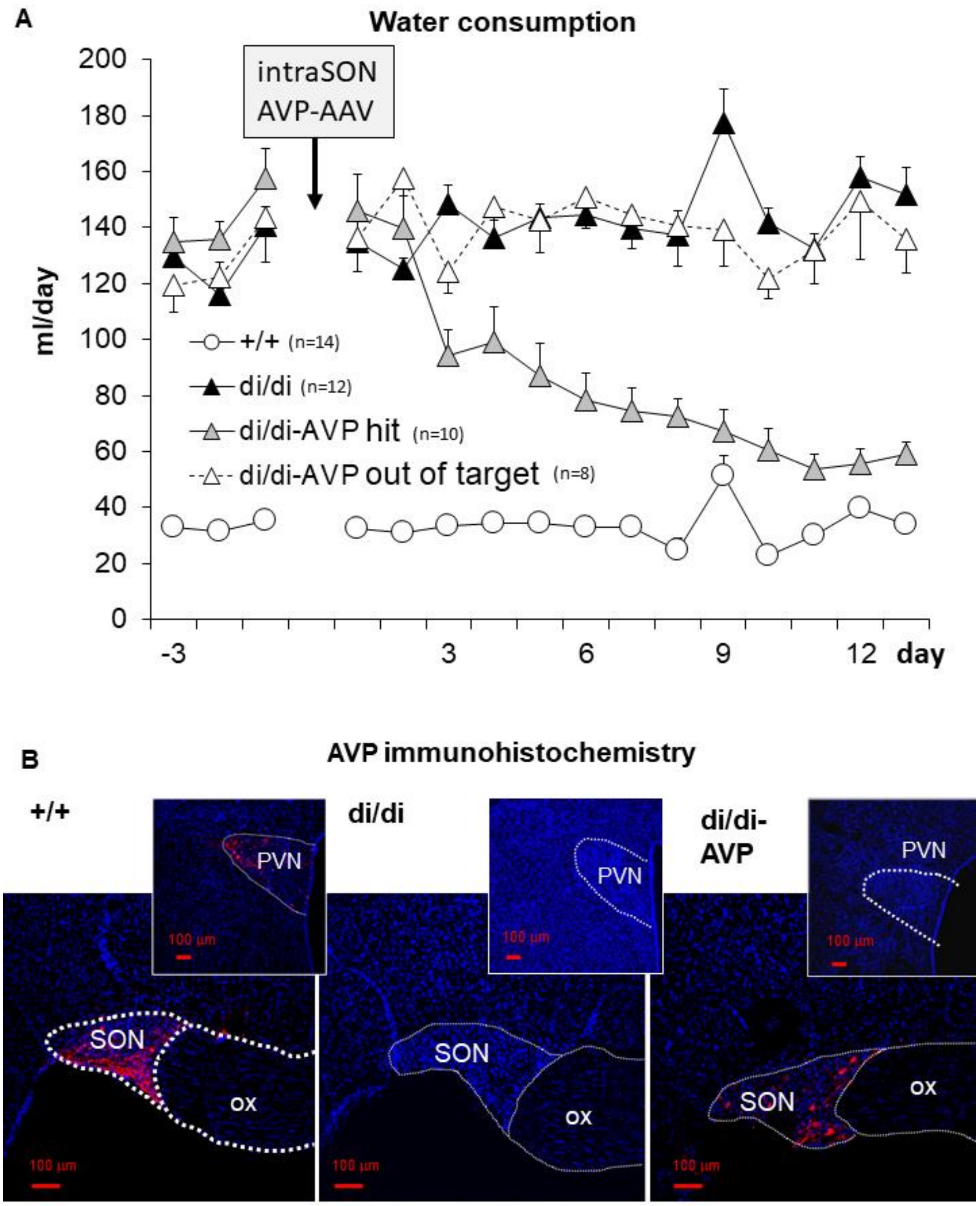

Figure S1. Confirmation of virus-driven rescue of AVP synthesis in the SON of male Brattleboro rats. (**A**) In line with previous results [25, 26] we confirmed that the high water consumption (mean ± SEM) of di/di rats was significantly reduced in a subset of AVP-AAV treated animals. The AVP-AAV animals showing no drop in their water consumption were considered as "no hits" and were excluded from later analysis. The criterion for selection of the "hits" was a positive reduction in water consumption calculated according to the following formula: 100-(average water consumption after operation)/(average water consumption before operation)*100. **(B)** Microphotographs showing immunohistochemistry for AVP in the brain of representative +/+, di/di and di/di-AVP animals. In the latter AVP synthesis was restored by AVP-AAV treatment in the SON, but not in the PVN. Dotted lines are used to delineate the respective nucleus from the surrounding tissue. Abbreviations: AVP, vasopressin; AVP-AAV, adenoassociated viral vector containing vasopressin sequence; di/di, vasopressin-deficient Brattleboro rat; di/di-AVP, di/di animals with vasopressin synthesis rescue in the SON; OX, optical chiasm; PVN, paraventricular nucleus of the hypothalamus; SON, supraoptic nucleus.

Table S1. Body weight change during 14 days after AVP-AAV injection into the SON.

	+/+	di/di	di/di-AVP good	di/di-AVP bad
Body weight gain (g)	26.7±2.87<br>n=14	32.8±5.33<br>n=12	68.2±5.40*#<br>n=9	54.5±4.28*#$<br>n=8

Statistical analysis revealed significant difference between groups ($F(3,39) = 18.16$, $p < 0.01$). * $p < 0.01$ significant difference from +/+, # $p < 0.01$ significant difference from di/di, $ $p < 0.01$ significant difference from di/di-AVP good.

Table S2. Individual resting ACTH and corticosterone levels in animals from Series 1 measured immediately before injection of egg white into the jugular vein.

Number	Group	ACTH (fmol/ml)	Corticosterone (pmol/ml)
9	+/+	20.1	*
10	+/+	1.5	239.2
12	+/+	4.6	385.2
13	+/+	3.7	386.9
15	+/+	1	119.3
16	+/+	1	24
17	+/+	1	168
37	+/+	1	114.4
39	+/+	1	*
40	+/+	2.8	331.2
1	di/di	0.7	36.5
7	di/di	*	228.7
31	di/di	1	22.5
32	di/di	1	134.9
33	di/di	1	73.7
34	di/di	12	*
35	di/di	2.7	108.5
36	di/di	1	280
41	di/di	*	17
51	di/di	0.4	106.5

3	di/di-AVP	3.7	166
22	di/di-AVP	0.5	16.8
24	di/di-AVP	1.3	11.5
25	di/di-AVP	13.6	294.3
26	di/di-AVP	1	230.4
29	di/di-AVP	1	192.5
43	di/di-AVP	1	112.4
44	di/di-AVP	1.3	68.2

*Due to technical reasons we could not detect both ACTH and corticosterone in all samples. No significant differences between the groups have been detected.

Table S3. Protocols for the immunohistochemical staining of the brain slices.

Protein/ Peptide	Permeabilization	Blocking	Primary antibody	Secondary antibody
AVP	1 h, 0.5% Triton-X 100 + 2% BSA	no	72 h, a-AVP 1:100 000 in 2% BSA	1.5 h, a-rabbit Alexa 594 1:1000 in PBS
c-Fos	30 min, 0.5% Triton-X 100 and 0.5% H_2O_2 in PBS	1 h, 2% BSA	48 h, at 4°C a-c-Fos 1:5000 in PBS	1h a-rabbit biotinylated 1:500 at RT<br>1.5 h ABC 1:1000 in 0.05 M Tris buffer followed by 0.2 mg/ml DAB, 0.1% nickel–ammonium sulfate and 0.003% H_2O_2 in Tris buffer

Abbreviations: a-: anti, ABC: avidin–biotin complex, AVP: vasopressin, BSA: bovine serum albumine, DAB: 3′-diaminobenzidine tetrahydrochloride, H_2O_2: hydrogen peroxide, PBS: phosphate-buffered saline, RT: room temperature,

Table S4. Sequences used to the design of the primer pairs for RT-PCR.

**Protein**	**forward sequence**	**reverse sequence**
RPS18	TTCAGCACATCCTGCGAGTA	TTGGTGAGGTCAATGTCTGC
POMC	GAGGTTAAGGAGCAGTGACTAAG	CGTCTATGGAGGTCTGAAGC
V1b-R	CTCTGCCGGGCTGTCAAGTA	ATGGCCAGCAGCATGTAAGT
CRH-R1	TCGGGAGAAGGCTACCAGAC	CCTGGATCGCTCCGACATC

Abbreviations: *RPS18: ribosomal protein S18*; POMC: proopiomelanocortin, the precursor of the adrenocorticotropin; V1b-R: the main AVP receptor on the anterior lobe of the pituitary; CRH-R1: the main corticotropin-releasing hormone receptor on the anterior lobe of the pituitary.
